# Saikosaponin D exerts antidepressant effect by regulating Homer1-mGluR5 and mTOR signaling in a rat model of chronic unpredictable mild stress

**DOI:** 10.1186/s13020-022-00621-8

**Published:** 2022-05-24

**Authors:** Chen-Yue Liu, Jian-Bei Chen, Yue-Yun Liu, Xue-Ming Zhou, Man Zhang, You-Ming Jiang, Qing-Yu Ma, Zhe Xue, Zong-Yao Zhao, Xiao-Juan Li, Jia-Xu Chen

**Affiliations:** 1 School of Traditional Chinese Medicine, Beijing University of Chinese Medicine, Beijing, 100029 China; 2 Formula-Pattern Research Center, School of Traditional Chinese Medicine, Jinan University, Guangzhou, 510632 China; 3 Institute of Chinese Materia Medica, China Academy of Chinese Medical Sciences, Beijing, 100700 China; 4 School of Basic Medical Sciences, Heilongjiang University of Chinese Medicine, Haerbin, 150040 China

**Keywords:** Saikosaponin D, Chronic unpredictable mild stress, Homer1-mGluR5, mTOR

## Abstract

**Background:**

Many studies about depression have focused on the dysfunctional synaptic signaling in the hippocampus that drives the pathophysiology of depression. *Radix Bupleuri* has been used in China for over 2000 years to regulate liver-qi. Extracted from *Radix Bupleuri*, Saikosaponin D (SSD) is a pharmacologically active substance that has antidepressant effects. However, its underlying mechanism remains unknown.

**Materials and methods:**

A chronic unpredictable mild stress (CUMS) paradigm was used as a rat model of depression. SD rats were randomly assigned to a normal control (NC) group or one exposed to a CUMS paradigm. Of the latter group, rats were assigned to four subgroups: no treatment (CUMS), fluoxetine-treated (FLU), high-dose and low-dose SSD-treated (SSDH and SSDL). SSD was orally administrated of 1.50 mg/kg and 0.75 mg/kg/days for three weeks in the SSDH and SSDL groups, respectively. Fluoxetine was administrated at a dose of 2.0 mg/kg/days. SSD’s antidepressant effects were assessed using the open field test, forced swim test, and sucrose preference test. Glutamate levels were quantified by ELISA. Western blot and immunochemical analyses were conducted to quantify proteins in the Homer protein homolog 1 (Homer1)-metabotropic glutamate receptor 5 (mGluR5) and mammalian target of rapamycin (mTOR) pathways in the hippocampal CA1 region. To measure related gene expression, RT-qPCR was employed.

**Results:**

CUMS-exposed rats treated with SSD exhibited increases in food intake, body weight, and improvements in the time spent in the central are and total distance traveled in the OFT, and less pronounced pleasure-deprivation behaviors. SSD also decreased glutamate levels in CA1. In CA1 region of CUMS-exposed rats, SSD treatment increased mGluR5 expression while decreasing Homer1 expression. SSD also increased expressions of postsynaptic density protein 95 (PSD95) and synapsin I (SYP), and the ratios of p-mTOR/mTOR, p-p70S6k/p70S6k, and p-4E-BP1/4E-BP1 in the CA1 region in CUMS-exposed rats.

**Conclusions:**

SSD treatment reduces glutamate levels in the CA1 region and promotes the expression of the synaptic proteins PSD-95 and SYP via the regulation of the Homer1-mGluR5 and downstream mTOR signaling pathways. These findings suggest that SSD could act as a natural neuroprotective agent in the prevention of depression.

**Supplementary Information:**

The online version contains supplementary material available at 10.1186/s13020-022-00621-8.

## Highlights

- Saikosaponin D, a pharmacologically active substance isolated from the Radix *Bupleuri* has been shown to have antidepressant effects.
- Its effects are mediated through the Homer1-mGluR5 and mTOR pathways.
- The findings support saikosaponin D’s actions as a neuroprotective agent.

**Supplementary Information:**

The online version contains supplementary material available at 10.1186/s13020-022-00621-8.

## Introduction

The effects of depression are widespread, as it disrupts productivity, impairs social interaction and overall health, reduces life expectancy, and increases the burden on the healthcare system [1]. In the last ten years, there has been growing concern regarding the impact of depression on global health, especially in low-income countries. Furthermore, the World Health Organization has predicted that depression will be the most disabling disease in the world by 2030 [2], highlighting the need for more studies to understand the pathophysiology of the disease and develop novel therapeutic regimens for depression that have fewer side effects. Among the studied compounds, Saikosaponin D (SSD) is being increasingly recognized due to its neuroprotective effects [3–5].

*Radix Bupleuri,* the root of *Bupleurum chinense* DC. or *Bupleurum scorzonerifolium* Willd., is a common component of Chinese herbal prescriptions such as Xiaoyaosan [6] and Chaihu-Shugan-San [7], which are administered clinically to improve symptoms of depression*.* Saikosaponin D (SSD) is a pharmacologically active ingredient extracted from *Radix Bupleuri* [8, 9], which is capable of ‘dispersing stagnated liver qi for relieving qi stagnation’ according to traditional Chinese Medicine (TCM) theory. Several researches reported that SSD was founded to have the effect to inhibit neuronal apoptosis and microglia activation as well as neuroinflammation in LPS-induced mice [3, 5]. These studies suggest that SSD could combat depression-like behaviors caused by inflammation. However, there are few studies on the effect of SSD on chronic stress-induced depression, and the underlying mechanism is still unclear. Chronic stress is closely related to neurological disorders. CUMS is a common rodent stress model that is frequently used in modern research investigating depression [10]. Katz and colleagues first reported the CUMS model [11]. Therefore, it is of important meaning to research the underlying mechanism of SSD in CUMS-induced rats in order to develop further therapeutic options for depression.

Recent clinical as well as basic research studies have linked the pathogenesis of depression with synaptic dysfunction in the prefrontal cortex and hippocampus [12], specifically within the hippocampal CA1 region. Chronic stress affects the hippocampus, a key region containing marginal structures involved in the processing of memories and emotional information. Neurological diseases can be caused by damage to the hippocampal CA1 region [13], which is also one of the brain regions most often studied in rodent models of depression [14, 15]. Furthermore, there is new evidence suggesting that Chinese herbal medicines may have antidepressant benefits resulting from their ability to upregulate the certain synaptic proteins expression [16–18]. Meanwhile, an increasing body of evidence suggests that Homer protein homolog 1 (Homer1)-metabotropic glutamate receptor 5 (mGluR5) and downstream mammalian target of rapamycin (mTOR) signaling pathways affect the expression of proteins involved in the modulation of synaptic plasticity that may drive the pathophysiology of depression. The pathogenesis of and the therapy to treat mood disorders are dependent on changes in mGluRs. For example, antidepressant-like behavior is induced by mGluR5 knockout [19], consistent with the mGluR5^−/−^mice, an antidepressant-like phenotype mice [20]. Located in the post-synaptic density, Homer1, a postsynaptic scaffold protein serves as a prominent link between group I mGluRs and downstream targets [21, 22]. It is expressed in two main isoforms, namely Homer1a and Homer1b/c isoforms, the latter of which is constitutively expressed, maintains synaptic transmission, and dendritic spine structure [23]. Genome-wide association studies (GWAS) have shown that Homer1 is a key target for mood disorders and antidepressants [24, 25]. The mTOR signaling pathway is a downstream intracellular signaling pathway that regulates synaptic protein synthesis and contributes to synaptogenesis [26]. Previous studies have suggested that activation of mGluR5 subtypes is required for mTOR phosphorylation in the hippocampal CA1 region [27]. When activated, mTOR complex 1 (mTORC1) causes the phosphorylation and activation of p70S6 kinase (S6K) and eukaryotic translation initiation factor 4E binding protein 1 (4E-BP1), resulting in the upregulation of synaptic proteins [e.g., postsynaptic density protein-95 (PSD-95) and synapsin I (SYP)] and the production of new synapses [28, 29]. Although SSD has been reported to exhibit antidepressant capacity, the underlying mechanism of action remains unclear. Therefore, the aim of this study was to identify the signaling pathways that mediate the antidepressant effects of SSD. We hypothesized that SSD promotes synaptic protein production in CUMS-exposed rats through the modulation of the Homer1-mGluR5 and mTOR signaling pathways.

## Materials and methods

### CUMS model establishment

Sixty 7-week-old male Sprague–Dawley rats were provided by the Beijing Vital River Laboratory Animal Research Center [license No. SCXK (Beijing) 2016–0006]. All the rats were housed in the Beijing University of Chinese Medicine's specific pathogen-free clean animal facility [license No. SYXK (Jing) 2016–0038, conventional breeding]. All animal experiments were approved by the Institutional Animal Care and Use Committee of Beijing University of Chinese Medicine. The experiments were conducted in strict accordance with the recommendations of the U.S. National Institutes of Health's Guide for the Care and Use of Laboratory Animals.

After a week of adaptation, 48 rats were assigned to the CUMS group randomly. These rats were subjected to a variety of randomly ordered, low-intensity social and environmental stressors for an extended period of time over the course of 6 weeks as previously described [30]. The selected stressors and schedule of exposure are summarized in Table 1. The 12 remaining rats were assigned to the normal control (NC) group and were not exposed to any stressors.

**Table 1:** The schedule of stressors used in CUMS paradigm

Stressors	Days of CUMS experiments
24-h water deprivation	1,15,27
overnight illumination	2,16,25,36
24-h food deprivation	3,19, 33,37
5-min heat exposure at 45 °C	10,20,31,
17-h white noise exposure	4,13,22, 26,41
17-h cage tilt at 45 degrees from the horizontal	5,21,29,34,40
24-h moist padding exposure	6,11,17,38
1-min tail pinch	8,12,18,23,30
3-h physical restraint	9,24,32,39
17-h strange object exposure	7,14,28,35,42

### Drug administration

**Fig. 1:**
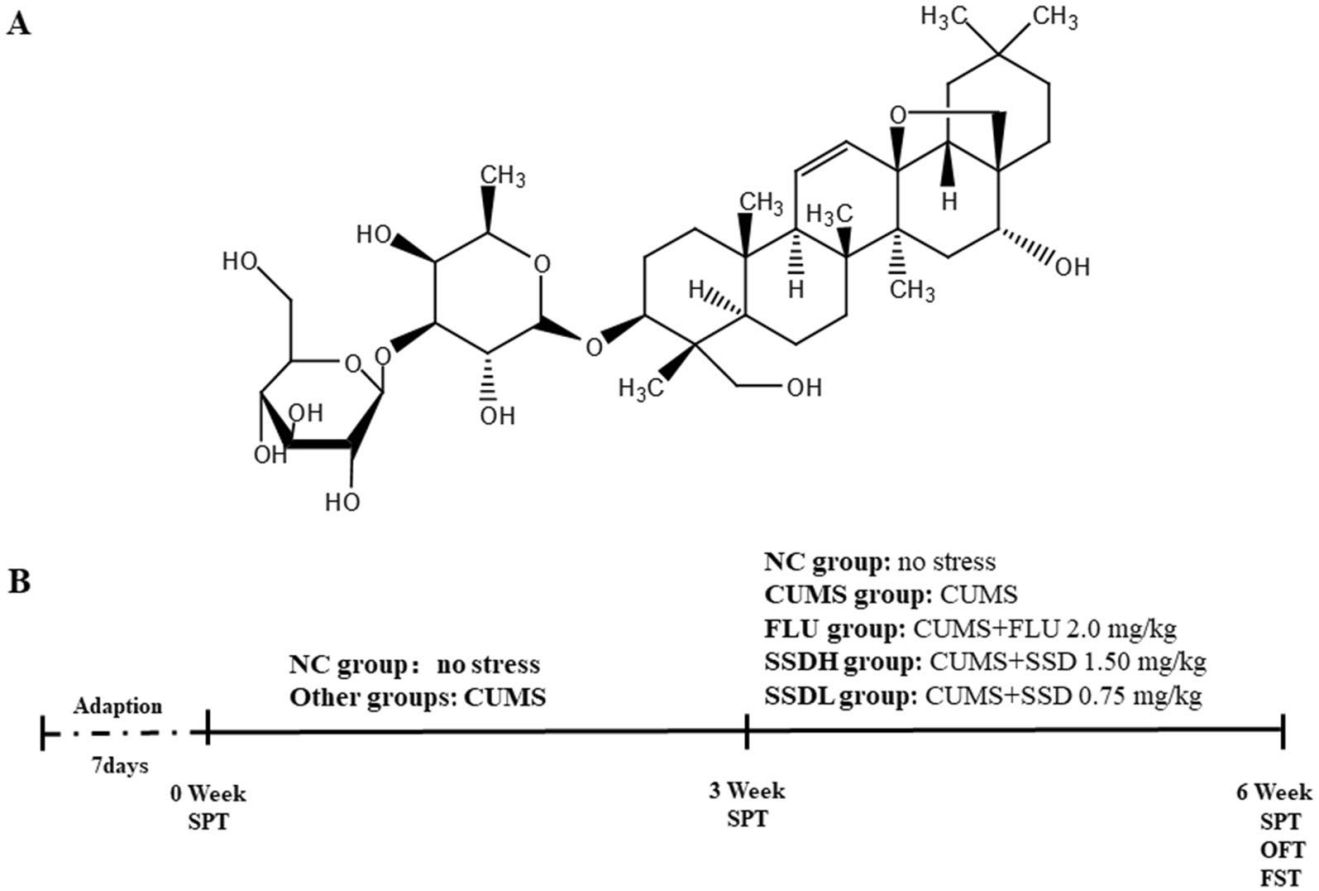
The molecular structure of SSD is shown in **A**. The experimental timeline is shown in **B**. The animals were provided a 7-day adaptation period prior to the start of the experiment. Except for the rats in the NC group, other rats were subjected to CUMS for six weeks and treated with different drugs from the 4^th^ week to the end

Three weeks after of exposed to CUMS, the CUMS-exposed rats were divided into four subgroups: a CUMS group (no drug treatment), a fluoxetine-treated group (FLU), and SSD high-dose (SSDH) and SSD low-dose (SSDL) groups. SSD was dissolved in dimethyl sulfoxide (DMSO) at a concentration of 0.1 percent. Drug administration was performed from the 4th week to the end. The rats in SSD high and low dose groups were treated with SSD (CAS Registry Number. 20874–52-6, PubChem CID: 107,793, 98% purity, batch number: B20150, Yuanye Bio-Technology, Shanghai, China) daily with 1.50 mg/kg and 0.75 mg/kg per day [31] by gavage, respectively. Rats were administered fluoxetine (2 mg/kg) in the FLU group, and rats received equal volumes of 0.1% DMSO by gavage in NC and CUMS groups. Figure 1A shows the SSD’s molecular structure. Figure 1B displays the experimental timeline.

### Behavioral testing

At the end of the drug treatment period, open field test (OFT), the sucrose preference test (SPT), and the forced swim test (FST) were conducted as previously described [32]. Details can be found in Additional file 1: Materials and Methods.

### Tissue sample collection

After a 12-h fasting period at the conclusion of the sixth week, six rats were randomly selected from each group. Rats were anesthetized with one percent sodium pentobarbital i.p., and arterially perfused with 4% paraformaldehyde. Then conventional paraffin embedding was performed. Using an Rat Brain Slicer Matrix (BSRAS001-1, ZIVIC Instrument, USA), the brains of the remaining 30 non-perfused rats were uniformly sectioned into coronal slices. Hippocampal CA1 tissue were isolated under an anatomical microscope.

### Enzyme-linked immunosorbent assays (ELISA)

Glutamate levels in each rats’ CA1 region were measured using an ELISA kit (Bio-Assay Systems, #EGLT-100).

### Quantitative real-time polymerase chain reaction (RT-qPCR)

The mRNA levels of mGluR5, Homer1, PSD-95 and SYP were quantified by RT-qPCR. RT-qPCR were performed as described [33] and detailed described in Additional file 1: Materials and Methods. The primer sequences shown in Table 2 were synthesized by Sangon Biotech Co., Ltd. (Shanghai, China).

**Table 2:** Forward and reverse primer sequences

Gene	Sequences
*GluR5*	F: CATGGAGCAGATCAGCAGCGTAG
	R: ATCAGGTAGGAGGAGCAGATTGGAG
*SYP*	F: GCTGTGTTTGCCTTCCTCTACTC
	R: TGATAATGTTCTCTGGGTCCGTG
*Homer1*	F: AGGAGAAGTCGCAGGAGAAGATGG
	R: CCTTGGCTCTGAGTTCTGTGTCAC
*PSD-95*	F: TCCAGTCTGTGCGAGAGGTAGC
	R: GGACGGATGAAGATGGCGATGG
*GAPDH*	F: CCATTCTTCCACCTTTGAT
	R: TGGTCCAGGGTTTCTTACT

### Western blot analysis

Homer1b/c, mGluR5, p-mTOR, mTOR, p70S6 kinase, p-p70S6 kinase, p-4E-BP1, 4E-BP1, PSD-95 and SYP expressions in hippocampal CA1 region of experimental rats were detected by Western blot analysis according to the protocol [6]. Details are listed at Additional file 1: Materials and Methods.

### IHC

The brain tissue was embedded in paraffin and cut into 5 μm sections for IHC experiment as described [6]. See Additional file 1: Materials and Methods for more details.

### Statistical analysis

Statistical analyses were performed using Statistical Package for the Social Sciences (SPSS) software (version 22.0; IBM Corp., Armonk, New York, NY, USA). Statistical significance was defined as *P* < 0.05. See Additional file 1: Materials and Methods for more details.

## Results

### SSD administration increases the body weight and food intake of CUMS rats

**Fig. 2:**
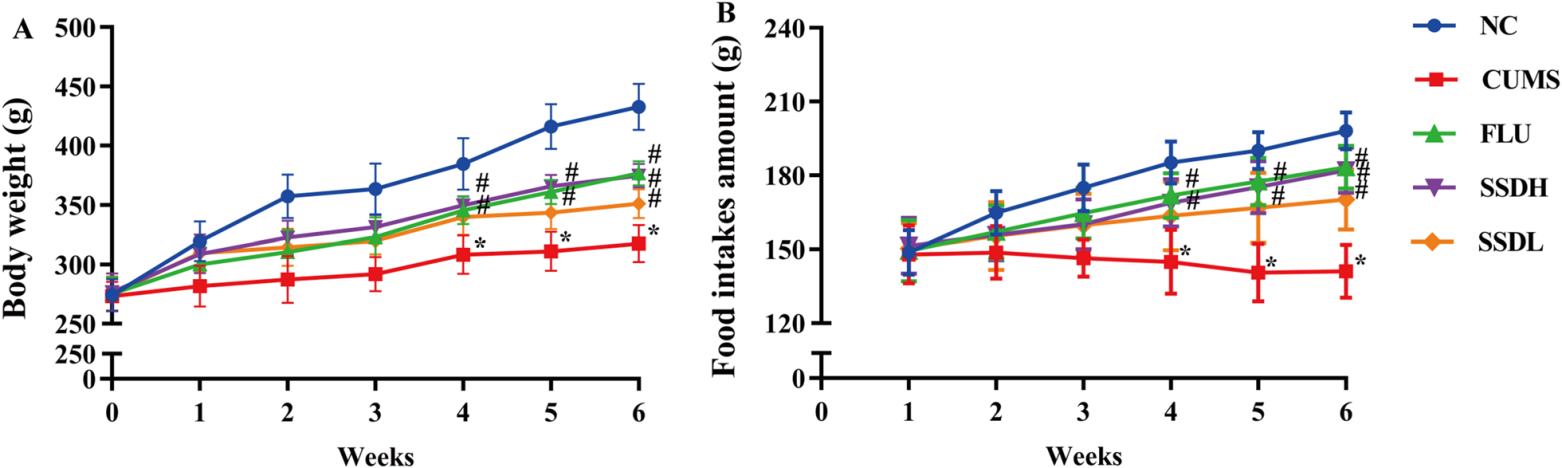
Changes in body weight and food intake in the CUMS rats. **A** Changes in body weight from week 0 to week 6. **B** Changes in food intake from week 1 to week 6. The data are expressed as the mean ± SD. ^*^P < 0.05 compared to the control group, ^#^*P* < 0.05 compared to the CUMS group; n = 12. CUMS, chronic unpredictable mild stress; SD, standard deviation

A rat model of CUMS (Fig. 1 and Table 1) was established over a 6-week period, throughout which weekly body weight and food intake were assessed. At week 0, there was no statistically significant differences in body weight among all groups of rats, as shown in Fig. 2A. The weekly body weight of rats in the CUMS group significantly decreased compared with that of the NC group (*P* < 0.05) in the 4th, 5th, and 6th weeks. From week 4 to week 6, FLU, SSDH and SSDL treatment increased the rats’ body weight significantly compared with that of rats in the CUMS group (*P* < 0.05). Figure 2B shows that the food intake of the rats in the five different groups did not initially vary. However, from the second to the sixth week, there was a significant difference in food intake between the NC and CUMS groups (*P* < 0.05), and during weeks 4 ~ 6, the levels of food intake of the rats in the SSD and FLU groups were higher than that of the rats in the CUMS group (*P* < 0.05).

### SSD ameliorates depression-like behaviors of CUMS rats

**Fig. 3:**
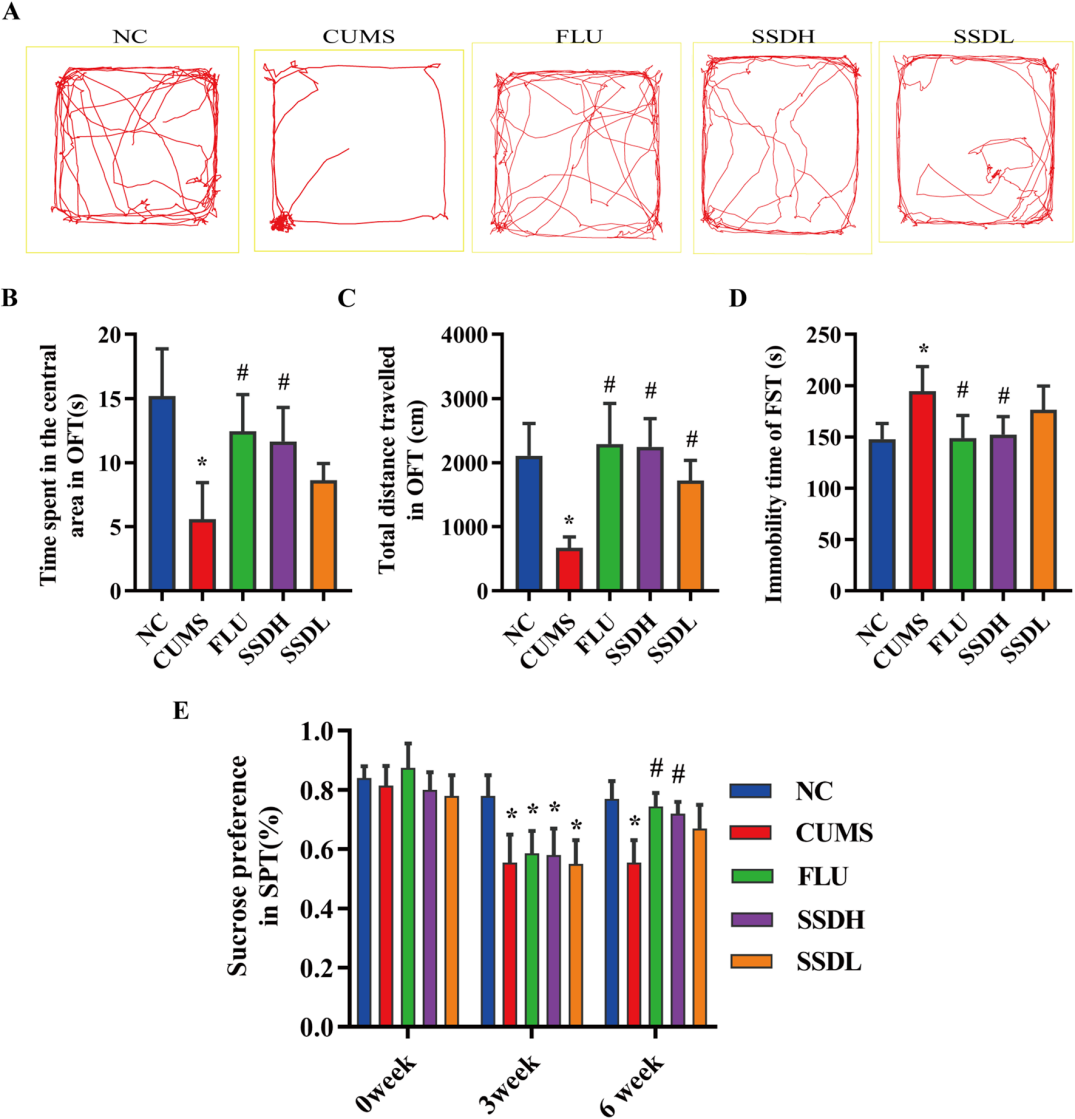
SSD ameliorates depression-like behaviors in CUMS-exposed rats. **A** Movement trajectory of the rats in each group. **B** The time spent in the central area of the OFT apparatus by each group of rats. **C** The total distance travelled by the rats in each group over a 5-min period in the OFT. **D** Immobility time of the rats in each group in the FST. **E** Sucrose preference as assessed by the SPT of the rats in each group at weeks 0, 3, and 6. All data are expressed as the mean ± SD. ^*^P < 0.05 compared to the control group, ^#^*P* < 0.05 compared to the CUMS group; n = 12

OFT, FST, and SPT were selected, as these are three classic behavioral tests for assessing depression-like behavior. Figure 3A shows the trajectory of the rats. The amount of time spent in the central region (Fig. 3B) was significantly shorter for the rats in the CUMS groups than in those of the NC group (*P* < 0.05). Rats in the FLU and SSDH groups spent more time in the central area than those of the CUMS group (*P* < 0.05). There was no significant difference in the time spent in the central area between the CUMS group and the SSDL-treated groups. The total distances travelled (Fig. 3C) by rats in the CUMS groups were shorter than those of the NC group (*P* < 0.05). Rats in the FLU, SSDH, and SSDL groups spent more time travelling in the central area than those in the CUMS group (*P* < 0.05). The SPT results are shown in Fig. 3D. The immobility times of rats in the CUMS groups were longer than that of the NC group (*P* < 0.05). Rats in the SSDH and FLU groups exhibited lower immobility times than rats in the CUMS group (*P* < 0.05). There was no significant difference in immobility time between rats in the CUMS and SSDL groups.

The SPT is the gold standard for evaluating decreases in pleasure in rodents. Anhedonia is characterized by a lack of interest in rewarding stimuli, which is a common symptom of affective disorders such as depression. The SPT was conducted on days 0, 21, and 41 (Fig. 3E). There was no significant difference in sucrose preference among the five groups at baseline. However, three weeks later, rats exposed to the CUMS paradigm (the CUMS, FLU, SSDH, and SSDL groups) exhibited a significant decrease in sucrose preference compared with that of the NC group (*P* < 0.05). This trend was reversed in the FLU and SSDH groups after three weeks of drug administration, with significantly higher sucrose preference rates compared with that of the CUMS group (*P* < 0.05). Therefore, a low dose of SSD is not as effective as a high dose in terms of the drug’s ability to improve depression-like behavior.

### SSD decreases glutamate levels in the hippocampal CA1 area of CUMS-exposed rats

**Fig. 4:**
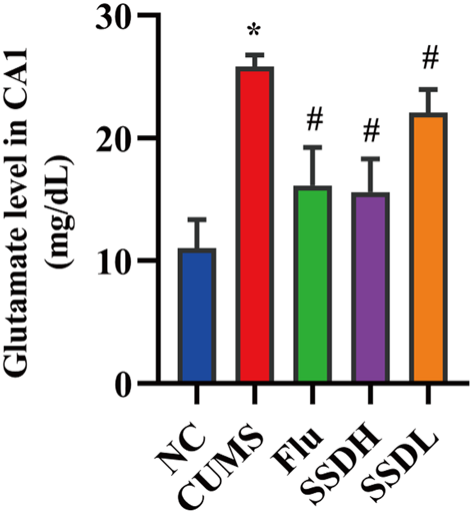
Glutamate levels in the hippocampus CA1 region of rats in each group. The data are expressed as the mean ± SD. ^*^P < 0.05 compared to the control group, ^#^*P* < 0.05 compared to the CUMS group; n = 12

As shown in Fig. 4, glutamate levels in the CA1 area of the rats in the CUMS group were significantly higher than those in the NC group (*P* < 0.05). This outcome was attenuated in the FLU, SSDH, and SSDL groups (*P* < 0.05).

### SSD regulates Homer1b/c and mGluR5 signaling in the hippocampal CA1 region of CUMS-exposed rats

**Fig. 5:**
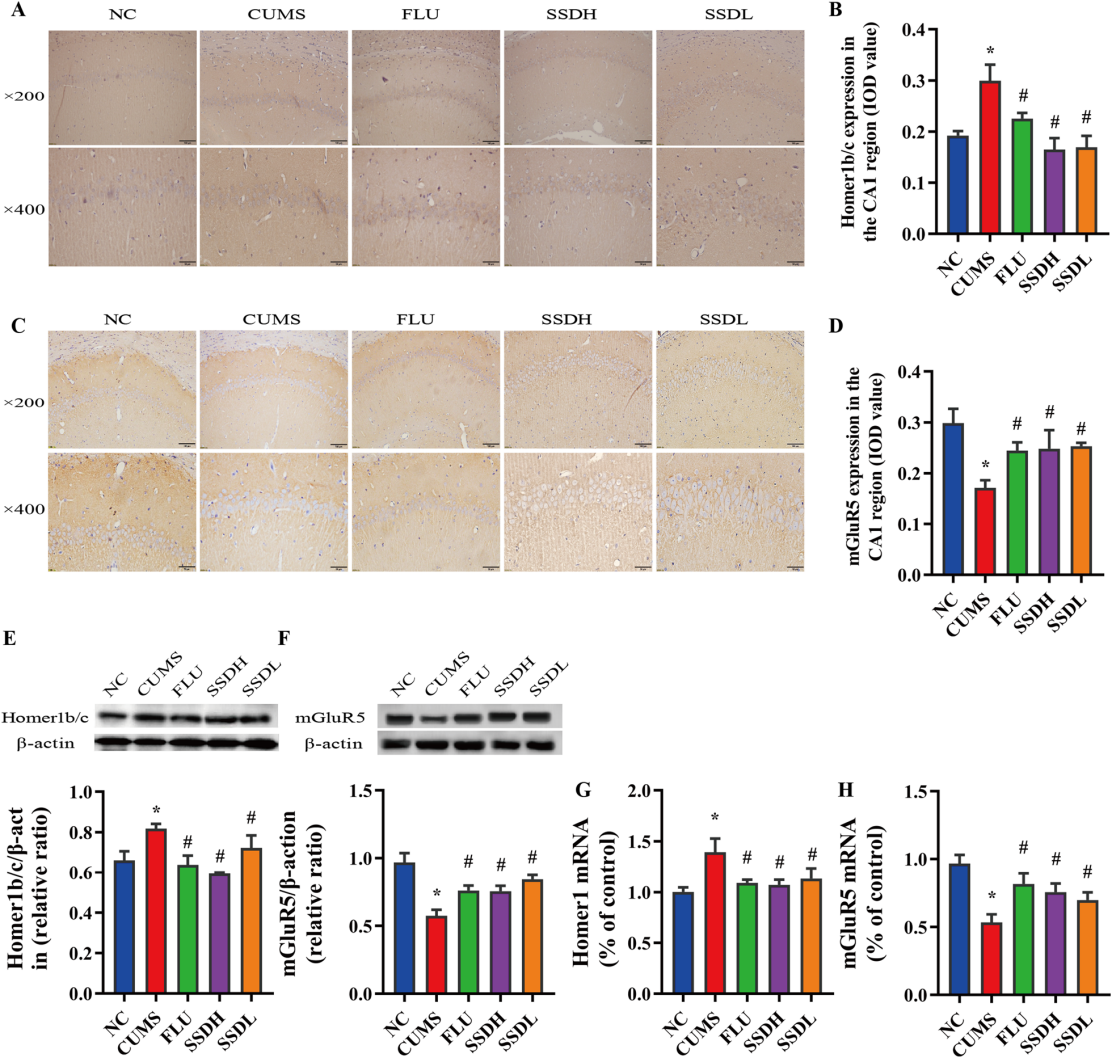
SSD treatment increases the expression of Homer1 and mGluR5 in the hippocampal CA1 area of the brains of CUMS-exposed rats. Images of IHC labeling at the original magnification (× 200 and × 400) and the respective IOD values of Homer1 (**A**,** B**) and mGluR5 (**C**,** D**) expression in the hippocampi of CUMS-exposed rats. (**E**,** F)** show representative western blot images and the relative ratios of Homer1 and mGluR5 expression in the hippocampal CA1 region of the different groups of rats exposed to the CUMS paradigm. (**G**, **H**) show the Homer1 and mGluR5 mRNA levels in the hippocampal CA1 region of the rats in each group. All data are expressed as the mean ± SD. ^*^P < 0.05 compared to the control group, ^#^*P* < 0.05 compared to the CUMS group; n = 6. SSD

In this study, we evaluated changes in Homer1b/c and mGluR5 expression in the hippocampal CA1 region of CUMS-exposed rats. IHC labeling revealed that the expression of Homer1b/c was significantly higher in the CUMS group than in the NC group, as illustrated in Fig. 5A and B (*P* < 0.05). In the FLU, SSDH, and SSDL groups, the expression of Homer1b/c in the hippocampal CA1 region was significantly higher than that of the CUMS group (*P* < 0.05). The results of the western blot (Fig. 5E) and RT-qPCR (Fig. 5G) experiments were consistent with the IHC results.

As shown in Fig. 5C and D, IHC labeling showed that the expression levels of mGluR5 in the hippocampal CA1 regions of rats in the CUMS group were reduced compared with those in the NC group (*P* < 0.05). This change was significantly attenuated in the FLU, SSDH, and SSDL groups, all of which exhibited higher levels of mGluR5 expression than that of the CUMS group in the hippocampal CA1 region (*P* < 0.05). The results of the western blot (Fig. 5F) and RT-qPCR (Fig. 5H) experiments were consistent with those of the IHC.

### SSD regulates the mTOR pathway in CUMS-exposed rats

**Fig. 6:**
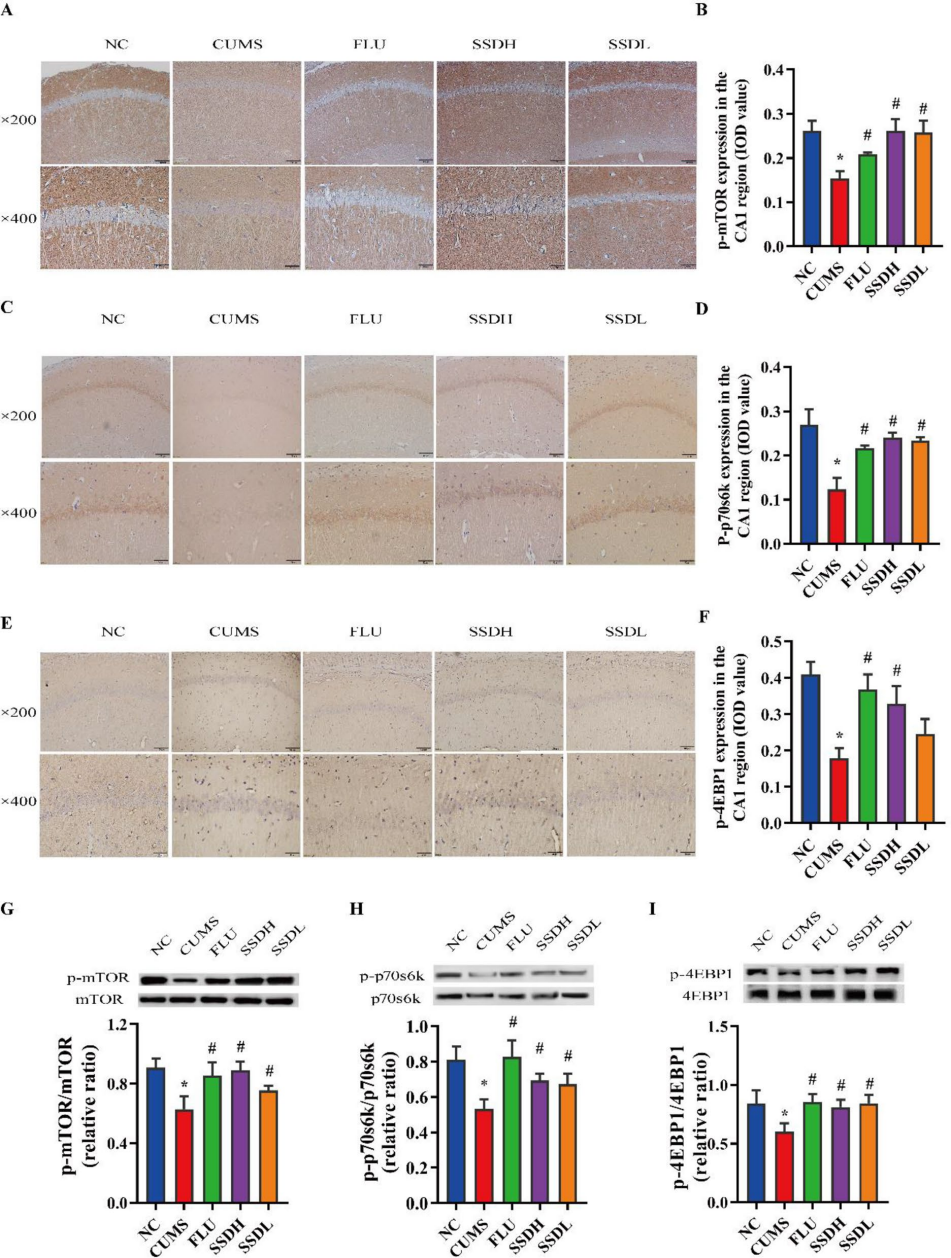
Treatment of CUMS-exposed rats with SSD results in elevated expression of p-mTOR, p-p70s6k, and p-4E-BP1 in the hippocampal CA1 region. IHC labeling at the original magnification (× 200 and × 400) and the respective IOD values of p-mTOR (**A**,** B**), p-p70s6k (**C**,** D**), and p-4EBP1(**E, F**) expression in the hippocampal CA1 region of CUMS rats. (**G**–**I**) show representative western blot images and the relative ratios of p-mTOR, p-p70s6k, and p-4EBP1 expression in the hippocampal CA1 regions of the different groups of CUMS-exposed rats. All data are expressed as the mean ± SD. ^*^P < 0.05 compared to the control group, ^#^*P* < 0.05 compared to the CUMS group; n = 6

Proteins and genes in the mTOR pathway were quantified in the hippocampal CA1 area of the rat brain by western blot, IHC labeling, and RT-qPCR to further investigate the probable mechanisms of action of SSD in CUMS-exposed rats. As shown in Fig. 6A, B, the expression of p-mTOR/ mTOR was significantly lower in the hippocampal CA1 area of rats in the CUMS group compared with that of the NC group (*P* < 0.05). Interestingly, after three weeks of SSD administration, the expression of p-mTOR in the hippocampal CA1 region was significantly upregulated (*P* < 0.05). The results of the western blot experiments showed that the ratio of p-mTOR/mTOR was decreased in rats of the CUMS group compared with that of rats in the NC group (*P* < 0.05). Figure 6C and D show that the level of expression of p-p70s6k was significantly reduced in the hippocampal CA1 area of the CUMS group compared with that of the NC group (*P* < 0.05). However, CUMS rats who were administered SSD exhibited increased p-p70s6k expression in the CA1 region of the hippocampus compared with that of CUMS-exposed rats who were not (*P* < 0.05). As shown in Fig. 6E and F, the level of expression of p-4E-BP1 was significantly lower in the hippocampal CA1 area of the rats in the CUMS group than in those of the NC group (*P* < 0.05). However, the expression of p-4E-BP1 in rats of the SSDH group was higher than that of the CUMS group (*P* < 0.05). The results of the western blot experiments evaluating the ratios of p-mTOR/mTOR (Fig. 6G), p-p70s6k/p70s6k (Fig. 6H) and p-4E-BP1/4E-BP1 (Fig. 6I) were consistent with the IHC results.

### SSD regulates PSD-95 and SYP expression in the hippocampal CA1 region of CUMS-exposed rats

**Fig. 7:**
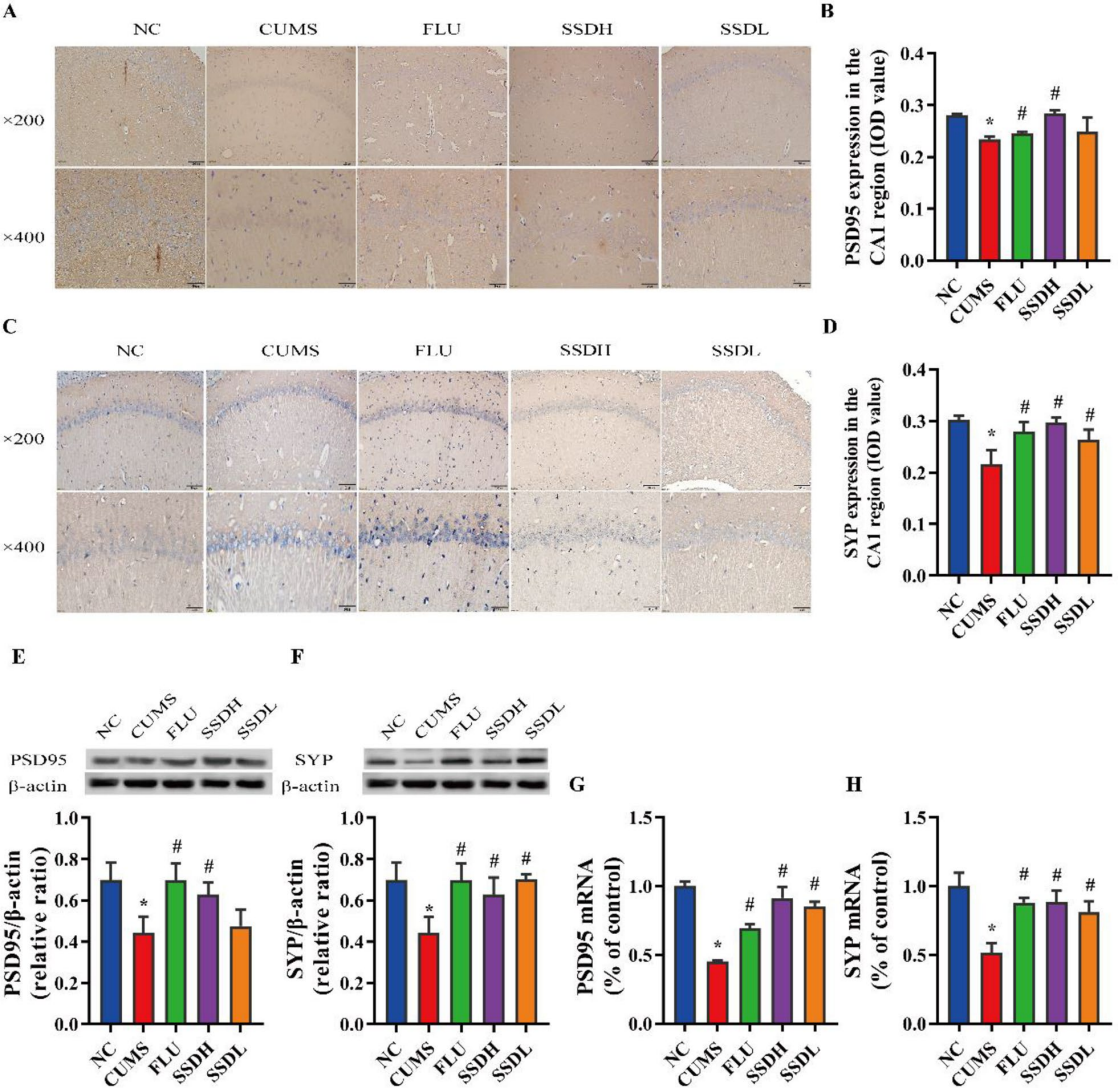
Treatment of CUMS-exposed rats with SSD results in elevated expression of PSD-95 and SYP in the hippocampal CA1 region. Images of IHC labeling at the original magnification (× 200 and × 400) and the respective IOD values of PSD-95 (**A**,** B**), and SYP (**C**,** D**) expression in the hippocampal tissue of CUMS-exposed rats. (**E**,** F)** show representative western blot images and the relative ratios of PSD-95 and SYP expression in the hippocampal CA1 regions of the different groups of CUMS-exposed rats. (**G**, **H**) show the PSD-95 and SYP mRNA levels in the hippocampal CA1 region of the rats in each group. All data are expressed as the mean ± SD. ^*^P < 0.05 compared to the control group, ^#^*P* < 0.05 compared to the CUMS group; n = 6

We investigated changes in the expression of the synaptic markers PSD-95 and SYP in the hippocampal CA1 region in CUMS-exposed rats. As shown in Fig. 7A and B, the IHC labeling revealed that the expression of PSD-95 in the hippocampal CA1 region of the rats in the CUMS group was significantly lower than that of the NC group (*P* < 0.05). Both FLU and SSDH treatment increased the expression of PSD-95 in the hippocampal CA1 region of rats compared with levels seen in the CUMS group (*P* < 0.05). There was no significant difference in the expression of PSD-95 between the CUMS and SSDL groups. The results of the western blot (Fig. 7E) and RT-qPCR (Fig. 7G) experiments were consistent with the IHC results. Figure 7C and D show that the expression levels of SYP in the hippocampal CA1 region of the rats in the CUMS group were decreased compared with levels in the NC group (*P* < 0.05). Both FLU and SSD administration significantly increased the expression of SYP in the hippocampal CA1 region of rats compared with that of the CUMS group (*P* < 0.05). The results of the western blot (Fig. 7F) and RT-qPCR (Fig. 7H) experiments were consistent with those of the IHC experiments.

## Discussion

**Fig. 8:**
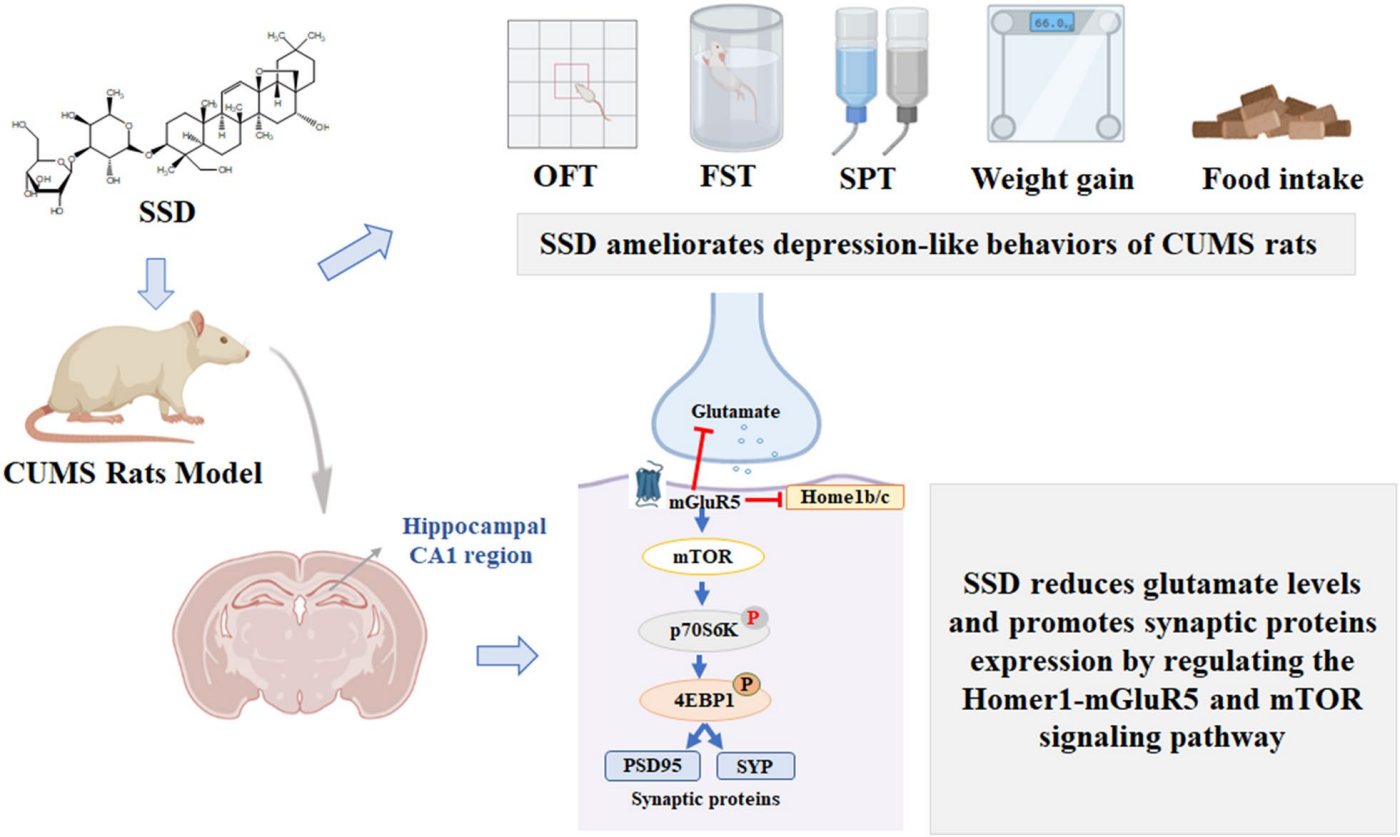
The potential mechanism of SSD against depression in CUMS rats

In this study, we made the following discoveries: (i) CUMS-exposed rats treated with SSD exhibited increased body weight and food intake, with a reduction in depression-like behaviors; (ii) SSD administration decreased glutamate levels in the rats’ CA1 region of CUMS-exposed; (iii) SSD treatment resulted in downregulated Homer1b/c expression as well as upregulated mGluR5, mTOR, PSD-95, and SYP expression, and increased p-mTOR/mTOR, p-p70S6k/p70S6k, and p-4E-BP1/4E-BP1 expression ratios in the CUMS-exposed rats’ CA1 region. From these results, we can conclude that synaptic damage caused by the elevated glutamate levels induced by CUMS could be reversed by SSD treatment. This amelioration of glutamate-induced synaptic damage appears to result from regulation of the Homer1-mGluR5 as well as downstream mTOR pathways (Fig. 8).

The CUMS, which is a common rodent stress model, involves the continuous and uninterrupted exposure of rodents to chronic stress environments; the chronic exposure to unpredictable micro-stressors in rats or mice leads to pronounced behavioral changes, including reduced responsivity to rewarding stimuli, and, clinically, the reduction of appetite [34]. There are behavioral correlations with core symptoms of depression [35]. FST, OFT and SPT were the commonly methods used in the research of depressive-like behavior in rodents. The FST it involves the exposure of animals to stress, which was shown to have a role in the tendency for major depression. The OFT can reflect an animal’s exploration characteristics and fear of a new environment, which can be used to quantitatively evaluate the animal’s spontaneous activity, exploration behavior, and the state of depression. SPT is an anhedonia test that measures sucrose consumption in rodents [36]. For decades, it has been known that stress is closely linked to the development of neurological disorders [11], and a previous study demonstrated that rats exposed to CUMS for 6 weeks developed depression-like behaviors [6]. LPS-induced mice produced depressive-like behaviors, including increased immobility time in FST, reduced movement distance and shortened stay time in the central region in the OFT, reduced sucrose consumption, which were consistent with depressive-like behaviors shown in this study [3, 5].

Study we explored, SSD administration improved depression-like behavior in rats exposed to CUMS. More specifically, rats in the SSDH and SSDL groups spent more time and travelled more than rats in the CUMS group in the OFT central area, and exhibited lower immobility time than those in the CUMS group. Loss of appetite is another important indicator of depression [34]. According to reports, 48 percent of individuals suffering from depression lose their appetite [37]. In the present study, SSD treatment to rats elevated both the food intake and body weight compared to that of rats who were exposed to the CUMS paradigm but did not receive SSD. Hong-Yan Li et al. as well as Bin Chao et al. found that SSD administration with UCMS (consistent with CUMS)-induced rats could improve depression-like behavior, which is consistent with the results of this study [4, 31].

SSD administration increased glutamate levels in the CUMS rats’ CA1 region, according to our current findings. Glutamate is considered to be the main excitatory neurotransmitter in the nervous system, exerting internal as well as external control over the information flow. There is evidence that abnormal glutamate synthesis, metabolism, and reabsorption by neurons in the brain are key components of the depression’s pathophysiological mechanism [38]. Furthermore, preclinical investigations have shown that CUMS increases glutamate levels in the extracellular of brain hippocampus region, resulting in glutamate-mediated excitotoxicity [39], which, in turn, leads to synaptic damage [40, 41]. Glutamate exposure also upregulates the expression of mGluR5 in the HT-22 hippocampal neuronal cell line [42]. Researches indicate that the dysregulation of glutamate transmission, primarily via changes in postsynaptic AMPARs, mGluRs and NMDAR, are important in the onset of psychiatric illnesses [43–45]. Thus, regulation of glutamate receptor activity will be a new target for novel antidepressant drugs [46]. Among these postsynaptic receptors, mGluRs have been shown to be closely related to the the pathological mechanism of mood disorders such as depression [47]. For example, antidepressant-like behaviors are induced by conditional knockdown of mGluR5 in gamma-aminobutyric acid- (GABA)ergic neurons, [19], which is congruent with mGluR5^−/−^mice, an antidepressant phenotype mice [20]. Overall, the results of this study show that mGluR5 is an important mediator of depression. Clinically [48], as well as in preclinical models of depression [49], decreased function of excitatory mGlu5 receptors should reduce the function of the glutamate system, which appears to be enhanced in depression. The results of this experiment are consistent with these studies that showed in depression models, there was a reduction of mGlu5R [50].

Homer1 is a postsynaptic scaffold protein, located in the postsynaptic density [21]. GWAS have revealed that Homer1 plays an important role mood disorders as well as antidepressant treatments [24, 25]. Researches also discovered that in China, rs7713917, the Homer1 gene variant, is a significant correlation to suicide attempts [51]. Homer1 has two distinct isoforms: the short isoform Homer1a and the long isoform Homer1b/c. Homer1b/c, which is constitutively expressed, maintains synaptic transmission and dendritic spine structure [23]. Homer1b/c has been shown to affect the response to antidepressants [52]. Therefore, in the present study, we concentrated on Homer1b/c. Previous research has found that chronic restraint stress increases Homer1b/c expressions in the hippocampus of rats [53]. Furthermore, the stress of social failure during youth increased the expression mRNA level of Homer1b/c in the dorsal mice’s hippocampus [54]. Some antidepressant treatments suppress depression-like behaviors by modulating Homer1 expression [55]. Thus, in this study, we assessed changes in Homer1b/c expression in the CUMS-exposed rats CA1 region.

The mTOR pathway has been shown to be regulated by the increase in Homer1 and the decrease in mGluR5 expression caused by chronic stress. Our findings show that mTOR induces the phosphorylation and activation of p70S6 and 4E-BP1, increasing the expression of synaptic proteins such as PSD-95 and SYP. Studies have also shown that antidepressants function by activating the mTOR pathway [28, 56]. mTOR is a serine/threonine protein kinase with a molecular weight of 289 kDa; it regulates autophagy, protein synthesis and lysosomal biogenesis [57–59]. Several studies have found that decreasing mTOR activation in animal depression models is beneficial. CUMS causes depressive-like behavior in mice and rats, and those changes are associated with decreased phosphorylation of mTOR and its downstream signaling components [60]. This is consistent with our findings. The activities of several receptors, including mGluRs, influence mTOR function [61–63]. Studies have suggested that the activation of mGluR5 subtypes is required for phosphorylation of mTOR in the CA1 area [27]. Once activated, mTORC1 causes the phosphorylation and activation of p70S6K and 4E-BP1, increasing the expression of the synaptic proteins PSD-95 and SYP that drive the formation of new synapses. Our data support these assumptions, as 3 weeks of SSD treatment significantly increased PSD-95 and SYP expression in the CA1 region of rats exposed to the CUMS paradigm. Although we initially confirmed the anti-depression effects and mechanism of SSD on CUMS rats, further researches are needed in vitro. Furthermore, pathway blockers and agonists need to be further validated in experiments. In summary, the findings suggest that chronic stress causes affective changes that can be attenuated by SSD treatment through the modulation of Homer1-mGluR5 and mTOR signaling in the hippocampus.

## Conclusion

In this study, we discovered that SSD treatment attenuated the increase in glutamate levels in the hippocampal CA1 region induced by chronic stress, which is known to lead to synaptic damage and promote the expression of synaptic proteins. Thus, SSD may alleviate depression-like behaviors in CUMS-exposed rats by regulating the Homer1-mGluR5 and mTOR signaling pathways. These findings point to the possibility that SSD could be administered as a natural neuroprotective agent in the prevention of depression.

### Supplementary Information

**Additional file 1.** Describes the Behavioral testing, RT-QPCR, Western blot analysis, Immunohistochemistry experimental procedure, and Statistical analysis in detail.

## Data Availability

The data used to support the findings of this study are included in the manuscript.

## Abbreviations

4E-BP1: Eukaryotic translation initiation factor 4E-binding protein 1
AMPAR: α-Amino-3-hydroxy-5-methyl-4-isoxazolepropionic acid receptor
CUMS: Chronic unpredictable mild stress
DMSO: Dimethyl sulfoxide
GABA: Gamma-aminobutyric acid
GAPDH: Glyceraldehyde-3-phosphate dehydrogenase
FLU: Fluoxetine
FST: Forced swim test
GWAS: Genome-wide association studies
Homer1: Homer protein homolog 1
IHC: Immunohistochemistry
IOD: Integrated optical density
mGluRs: Metabotropic glutamate receptors
mTOR: Mammalian target of rapamycin
mTORC1: MTOR complex 1
NC: Normal control
NMDAR: N-methyl-D-aspartate receptor
OFT: Open field test
RT-qPCR: Real-time quantitative polymerase chain reaction
SPT: Sucrose preference test
PSD-95: Postsynaptic density protein 95
PVDF: Polyvinylidene difluoride
S6K: P70S6 kinase
SSD: Saikosaponin D
SSDH: Saikosaponin D high-dose
SSDL: Saikosaponin D low-dose
SYP: Synapsin I
TBST: Tris-buffered saline containing 0.1% Tween 20
