# Correction: Saikosaponin D exerts antidepressant effect by regulating Homer1-mGluR5 and mTOR signaling in a rat model of chronic unpredictable mild stress

**DOI:** 10.1186/s13020-023-00845-2

**Published:** 2023-11-08

**Authors:** Chen-Yue Liu, Jian-Bei Chen, Yue-Yun Liu, Xue-Ming Zhou, Man Zhang, You-Ming Jiang, Qing-Yu Ma, Zhe Xue, Zong-Yao Zhao, Xiao-Juan Li, Jia-Xu Chen

**Affiliations:** 1 School of Traditional Chinese Medicine, Beijing University of Chinese Medicine, Beijing, 100029 China; 2 Formula-Pattern Research Center, School of Traditional Chinese Medicine, Jinan University, Guangzhou, 510632 China; 3 Institute of Chinese Materia Medica, China Academy of Chinese Medical Sciences, Beijing, 100700 China; 4 School of Basic Medical Sciences, Heilongjiang University of Chinese Medicine, Haerbin, 150040 China

**Correction: Chinese Medicine (2022) 17:60** **https://doi.org/10.1186/s13020-022-00621-8**

Following publication of the original article [1], the authors identified errors in Figs. 6A, C and 7A. In detail, the wrong images were used in the FLU and SSDL groups in Fig. 6C, as well as all groups in Figs. 6A and 7A. The errors were caused by a mistake in the layout and selection of representative images. The correct figures are shown as below.

**Fig. 6:**
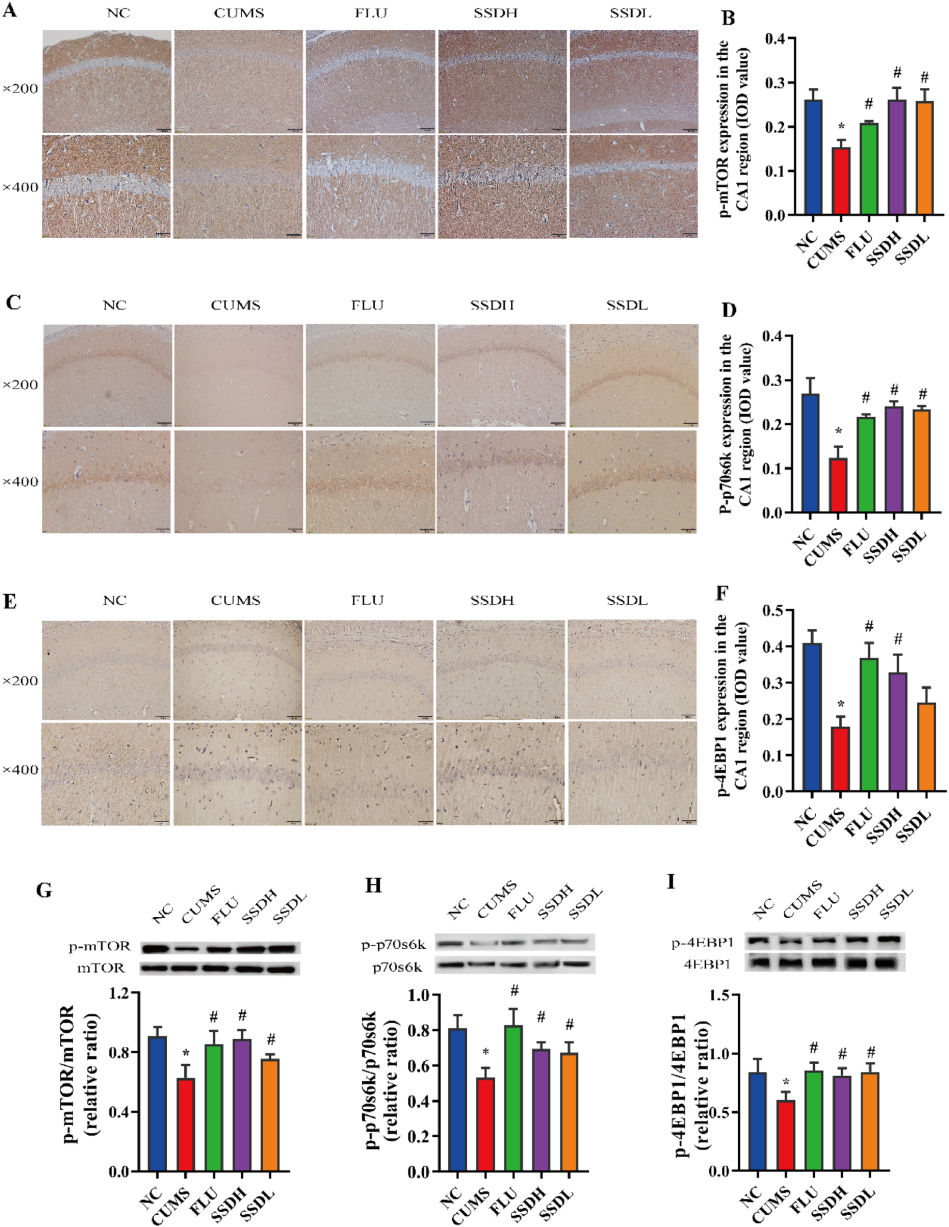
Treatment of CUMS-exposed rats with SSD results in elevated expression of p-mTOR, p-p70s6k, and p-4E-BP1 in the hippocampal CA1 region. IHC labeling at the original magnification (× 200 and × 400) and the respective IOD values of p-mTOR (**A**, **B**), p-p70s6k (**C**, **D**), and p-4EBP1(**E**, **F**) expression in the hippocampal CA1 region of CUMS rats. (G–I) show representative western blot images and the relative ratios of p-mTOR, p-p70s6k, and p-4EBP1 expression in the hippocampal CA1 regions of the different groups of CUMS-exposed rats. All data are expressed as the mean ± SD. *P < 0.05 compared to the control group, #P < 0.05 compared to the CUMS group; n = 6

**Fig. 7:**
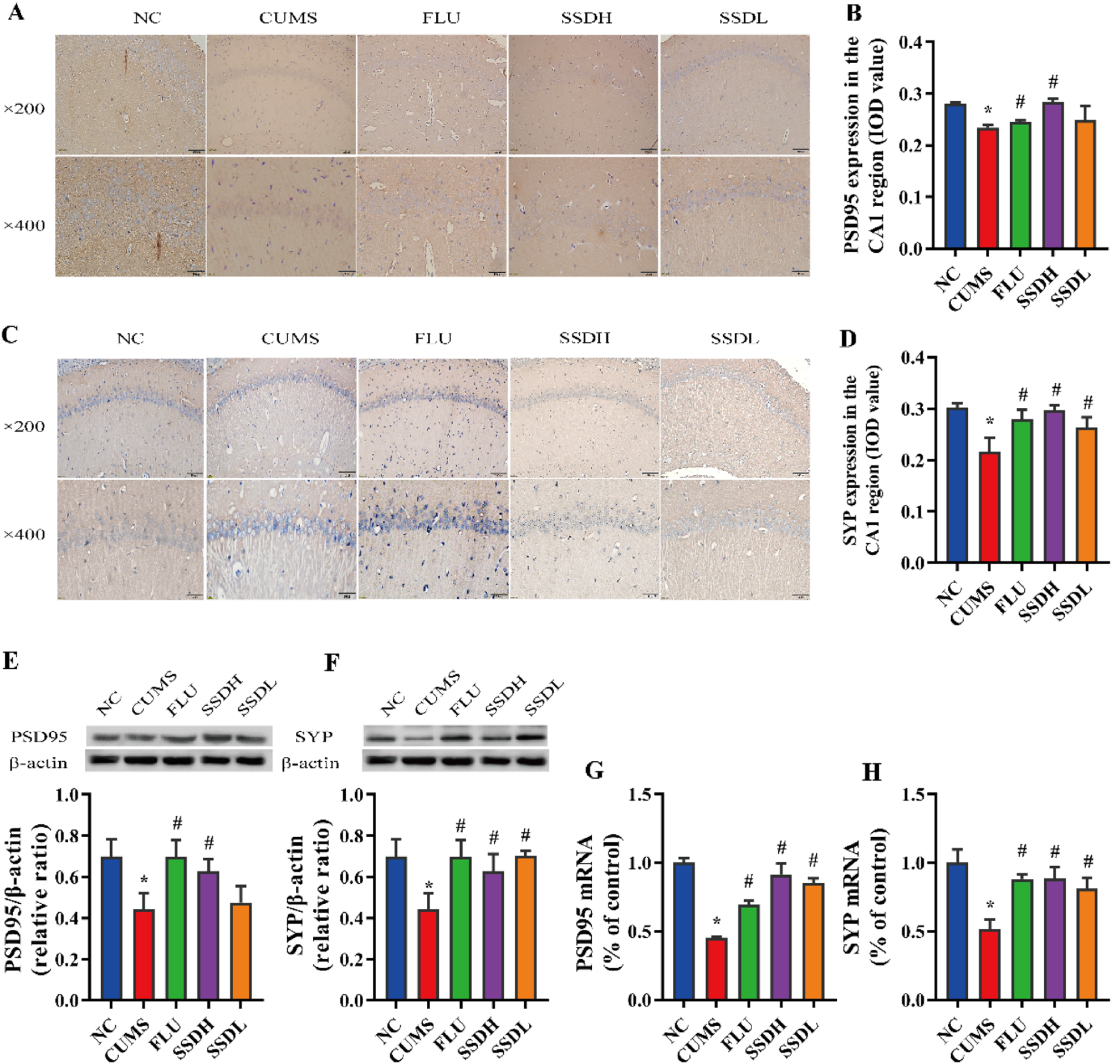
Treatment of CUMS-exposed rats with SSD results in elevated expression of PSD-95 and SYP in the hippocampal CA1 region. Images of IHC labelling at the original magnification (× 200 and × 400) and the respective IOD values of PSD-95 (**A**, **B**), and SYP (**C**, D) expression in the hippocampal tissue of CUMS-exposed rats. **E**, **F** show representative western blot images and the relative ratios of PSD-95 and SYP expression in the hippocampal CA1 regions of the different groups of CUMS-exposed rats. **G**, **H** show the PSD-95 and SYP mRNA levels in the hippocampal CA1 region of the rats in each group. All data are expressed as the mean ± SD. *P < 0.05 compared to the control group, #P < 0.05 compared to the CUMS group; n = 6

The authors apologize for the errors and state that this does not change the results and the scientific conclusions of this study. The original article [1] has been corrected.
