# Supplementary material for: Saikosaponin D exerts antidepressant effect by regulating Homer1-mGluR5 and mTOR signaling in a rat model of chronic unpredictable mild stress
Source: Chin Med. 2022 May 24;17:60. doi: 10.1186/s13020-022-00621-8 (PMC9128259; doi:10.1186/s13020-022-00621-8)
Supplement: Supplementary file 1 — Additional file 1. Describes the Behavioral testing, RT-QPCR, Western blot analysis, Immunohistochemistry experimental procedure, and Statistical analysis in detail. [file 13020_2022_621_MOESM1_ESM.docx]

**S1 Materials and Methods**

**Text**

**MATERIALS AND METHODS**

**Behavioral testing**

*OFT*

A 100×100×50 cm box constructed with black sidewalls and a floor was placed in a quiet room. All the rats from the different groups were placed in the testing room 30 minutes before the experiment for acclimatization. Each rat was gently placed in the central area of the box and allowed them to explore for five minutes. After every test session, 75% ethanol was used to clean the box. A video camera recorded the movement of the animals throughout the test period, and the software Noldus Vision 7.0 was used to track and measure the time spent in the central area and the total distance.

*SPT*

The SPT was carried out as previously stated. The testing was performed at baseline, on day 21, and the end. Briefly, on the first day, fill each cage with two bottles of one percent sucrose water, and let the rats drink freely. The next day,

distilled water and one percent sucrose solution were placed in identical looking bottles, weighed and given to the rats to drink freely. The formal trial began after the training. All the rats underwent a fasting period on the first day, with no intake of solid food or liquids. Then, distilled water and one percent sucrose solution were placed in identical looking bottles, weighed and given to the rats to drink for one hour and reweighed. Sugared water consumption is the percentage of sugared water consumed as a percentage of total liquid consumed.

*FST*

The FST was performed in accordance with our previous protocol. In brief, One rat from each group were gently placed in five water tanks (measuring 80 cm ×40 cm), filled with deionized water at the temperature of 23±1 °C. The movement of the rats in the tank was tracked for five minutes, and the time spent immobilized was recorded.

**Quantitative real-time polymerase chain reaction (****RT-qPCR)**

TRIzol reagent (Sigma) was used to extract RNA from the hippocampal CA1 region of rats, and cDNA was synthesized using a RevertAid First Strand cDNA Synthesis Kit (Thermo Scientific). RT-qPCR was conducted with a 20 mL volume of Power SYBR® Green PCR Master Mix (Thermo Fisher Scientific, USA) using a CFX96TM Real-Time System (Bio-Rad, USA) under the following cycling conditions: 95 °C for 10 minutes, followed by 40 cycles at 95 °C for 15 seconds and 56 °C for 1 minute. Relative levels of mRNA were quantified using Bio-Rad CFX Manager 2.1 (Bio-Rad, USA). The mRNA levels were normalized by glyceraldehyde-3-phosphate dehydrogenase (GAPDH). The 2^-△△Ct^ method was used to calculate the relative expression of mRNA.

**Western blot analysis**

Tissues from the hippocampal CA1 areas of experimental rats were homogenized in ice-cold radioimmunoprecipitation assay buffer containing phenylmethylsulphonyl fluoride (Beyotime Biotechnology, Shanghai, China). The supernatant was collected, and proteins were quantified using a bicinchoninic acid protein assay kit (Thermo Scientific, USA). A total of 45 μg of samples were separated by 10% sodium dodecyl sulfate-polyacrylamide gel electrophoresis before being transferred to polyvinylidene difluoride (PVDF) membranes. After blocking with 5% defatted milk in TBST (tris-buffered saline containing 0.1% Tween 20) for 1 h at room temperature, the samples were incubated in primary antibody solutions overnight at 4 °C [anti-Homer1b/c (Santa Cruz, sc-25271 1:2,000), anti-mGluR5 (Millipore, AB5675, 1:1,000), anti-p-mTOR (Santa Cruz, sc-293133, 1:2,000), anti-mTOR (Cell Signaling, 2983, 1:2,000), anti-p70S6 kinase (Cell Signaling, 34475, 1:1000), anti-p-p70S6 kinase (Thermo Fisher, PA5-37733, 1:2,000), anti-PSD-95 (Abcam, ab238135, 1:2,000), anti-p-4E-BP1 (Cell Signaling, 28551, 1:4,000), anti-4E-BP1(Abcam, ab32024, 1:2,000)，and anti-SYP (Abcam, ab 254349, 1:1,000)]. After incubation, TBST was used to wash the PVDF membranes, and samples were subsequently incubated with a horseradish peroxidase-conjugated secondary antibody solution (1:5,000). Samples were washed with TBST three times, and the signals were detected using an enhanced chemiluminescence solution and an Azure Bioimaging system (California, USA). β-actin (1:5,000) was used as an internal control. The results were quantified using ImageJ software (U.S. National Institutes of Health, Bethesda, MD, USA).

**Immunohistochemistry (IHC)**

5 μm paraffin-embedded brain tissue samples were defatted with xylene and dehydrated with graded ethanol (100–50 percent) for 4 hours in an oven at 60 °C. The slides were subsequently boiled in a microwave oven for 10 min, washed three times in TBST, and incubated in 3% H_2_O_2_ to quench endogenous peroxidase activity (30 min at RT). The tissue sections were then incubated in a primary antibody solution [anti-mGluR5 (1:100), anti-p-4E-BP1 (1:200), anti-PSD-95 (1:200), anti-p-mTOR (1:300), anti-Homer1 (1:200), anti-p-p70S6K (1:200), and anti-SYP (1:300)] at 4 ℃ overnight. The next day, all slides were incubated for 30 min at 37 °C in a solution containing secondary antibodies (Beijing Biosynthesis Biotechnology Co. Ltd., China), followed by hematoxylin staining. 3,3’-diaminobenzidine was used as a chromogen. The sections were photographed using an Olympus BX53 microscope. Integrated optical density (IOD) was analyzed using Image-Pro Plus 6.0 software (Rockville, USA).

**Statistical analysis**

The mean standard deviation (SD) is used to express all data obtained from the experiments. Data with a normal distribution and homogenous variances were analyzed by one-way analysis of variance (ANOVA). Dunnett’s T3 test was used to analyze data with a normal distribution but no homogeneity of variance. Nonparametric analysis was used to analyze data that was not normally distributed. Body weight and food intake data were compared using repeated-measures ANOVAs to detect significant differences when time and stress were taken into account.
